# The impact of the COVID-19 pandemic on symptomatic breast cancer presentations in an Irish breast cancer unit: a retrospective cohort study

**DOI:** 10.1007/s11845-024-03688-4

**Published:** 2024-04-19

**Authors:** Áine Higgins, Seamus O’Reilly, Martin J. O’Sullivan

**Affiliations:** 1 Department of Breast Surgery, Cork University Hospital and University College Cork, Cork, Ireland; 2 Department of Medical Oncology, College of Medicine and Health, Cork University Hospital and Cancer Research@UCC, University College Cork, Cork, Ireland; 3 Department of Breast Surgery, College of Medicine and Health, Cork University Hospital and Cancer Research@UCC, University College Cork, Cork, Ireland

**Keywords:** Breast cancer, COVID-19, Metastatic cancer, Pandemic, Symptomatic breast cancer, Tumour characteristics

## Abstract

**Background:**

The coronavirus-19 (COVID-19) pandemic caused delays in the diagnosis and management of breast cancer which may have affected disease presentation. The aim of this study was to compare rates of metastatic disease, tumour characteristics and management in breast cancer patients diagnosed before and after the onset of COVID-19.

**Methods:**

A retrospective chart review was conducted on patients in a university teaching hospital who were diagnosed with invasive symptomatic breast cancer in 2019 (prepandemic control group) and in 2020, 2021, and 2022 (pandemic study groups). Rates of new metastatic presentations, tumour histopathological characteristics, operation type, and therapies administered were statistically compared.

**Results:**

A total of 1416 patients were identified. There was a significant increase in new metastatic breast cancer presentations in 2022 compared to 2019 (14.0% vs 3.8%, *p* ≤ 0.001), with non-significant increases in 2020 and 2021. Rates of adjuvant radiotherapy increased in 2020 and decreased in 2022 compared to 2019, with no significant change in neoadjuvant or adjuvant chemotherapy rates. Rates of axillary surgery increased during 2020 and 2021. There was an increase in high-grade tumours and lymphovascular invasion (LVI), and less frequent oestrogen receptor (ER) positivity in pandemic groups. No significant change was noted in BCS to mastectomy ratios, overall nodal positivity rates, or median tumour size.

**Conclusion:**

Symptomatic breast cancers diagnosed since the onset of COVID-19 demonstrated an increase in new metastatic presentations and more aggressive histopathological characteristics when compared to a pre-pandemic control group. Rates of adjuvant radiotherapy and axillary surgery increased during the pandemic.

## Introduction

The first case of the COVID-19 virus was confirmed in Ireland on 26th February 2020 [1], with the first ensuing population-level lockdown ordered on 27th March 2020. Breast cancer services were disrupted at the onset of the pandemic due to the redeployment of staff and resources to care for COVID-19 patients, delays in elective diagnostic and surgical procedures, and staff shortages due to illness [2]. The pausing of BreastCheck, the Irish national breast screening programme, from March to October 2020 and January to March 2021 along with delays in symptomatic patients presenting to rapid access clinics due to fear of contracting the virus compounded the effect [3]. With symptomatic and screening services now restored to normal pre-pandemic activity, the longer-term impact of pandemic disruptions on Irish breast cancer patients is yet to be fully examined.

Data from several countries have demonstrated reductions from expected rates of breast cancer diagnoses in 2020 along with delays in time to surgery from diagnosis [4–15]. Preliminary data from the National Cancer Registry Ireland indicates a decreased number of breast cancer diagnoses when compared to predicted figures [16]. Modelling studies in the United Kingdom (UK) have projected an additional 7.9–9.6% additional deaths from breast cancer in the next 5 years [17]. This has led to the question of whether more advanced breast cancers have been diagnosed since the onset of the pandemic; the available literature indicates that this is the case [18–23], but the evidence is far from conclusive [24, 25]. As a result of differing approaches to the re-organisation of breast cancer care between countries and a focus on screen-detected cancers in available studies, further research to quantify the impact on symptomatic breast cancer patients is necessary.

The working hypothesis in the present study was that symptomatic breast cancer patients were presenting with more advanced disease since the onset of the COVID-19 pandemic. The aim of this retrospective cohort study was to identify patients who were diagnosed with breast cancer after presenting to a National Cancer Control Programme rapid access symptomatic breast cancer clinic before and after the onset of the pandemic and to compare disease presentation and management between these groups.

The objectives of the research were as follows:

1. To compare the rates of new diagnoses of stage IV breast cancer before and after the onset of COVID-19
2. To compare the rates of neoadjuvant and adjuvant therapies in patients surgically treated for invasive breast cancer before and after the onset of COVID-19
3. To compare prognostic factors including nodal positivity and tumour characteristics including pathological stage, histological grade, subtype, and size between symptomatic breast patients surgically treated for invasive breast cancer before and after the onset of COVID-19
4. To compare the breast-conserving surgery (BCS) to mastectomy ratio for invasive breast cancer in patients diagnosed before and after the onset of COVID-19

The relevance of this study is to highlight the importance of public health messaging around breast cancer and developing mitigation strategies to ensure continued access to screening and multi-disciplinary breast cancer care during future periods of strain on healthcare systems.

## Methods

### Patients

Consecutive patients who were diagnosed with invasive breast cancer presenting to the symptomatic breast clinic in Cork University Hospital in 2019 were identified as the pre-pandemic control group. All patients diagnosed with breast cancer in the same centre in 2020, 2021, and 2022 were identified as the pandemic study groups and were separately compared to the control group in terms of rates of stage IV disease at presentation, rates of neoadjuvant and adjuvant therapies, rates of nodal metastases, and rates of axillary surgery.

To compare tumour characteristics and BCS to mastectomy ratios, patients who were surgically treated for invasive breast cancer from March 1st to December 31st, 2019 were identified and separately compared to those treated from March 1st to December 31st in 2020, 2021, and 2022. Clinical, demographic, and pathological characteristics were extracted from postoperative histopathological reports and recorded by retrospective chart review. Patients who had neoadjuvant chemotherapy (NAC) were excluded from analysis of tumour characteristics.

### Variables

Rates of stage IV disease at presentation, nodal positivity, rates of NAC and adjuvant therapies, and axillary surgery rates were evaluated by reviewing histopathological and clinical records of breast cancer patients between 2019 and 2022, recording disease stage as per the American Joint Committee on Cancer (AJCC) 8th edition of the cancer TNM staging system [26].

Nodal status was recorded as positive or negative for metastases, with cases where isolated tumour cells were found classed as negative as per pathology reporting guidelines [27].

Post-operative histopathological reports were reviewed for each patient who had upfront surgical treatment for invasive breast cancer without NAC. Histological tumour size was recorded in centimetres, with the size of the largest focus recorded in cases of multicentric disease. The oestrogen receptor (ER), progesterone receptor (PR), and human epidermal growth factor receptor-2 (HER-2) status were recorded as positive or negative in each case.

Histological grades and subtypes were recorded for each patient, with subtypes grouped into invasive ductal cancer (IDC), invasive lobular cancer (ILC), invasive carcinoma with mixed ductal and lobular morphology, and other subtypes (including papillary, mucinous, and tubular). The type of operation (BCS or mastectomy) that each patient had as well as the presence or absence of lymphovascular invasion (LVI) were recorded. The pathological stage was recorded, again as per the cancer TNM staging system [26]. It is also worthwhile noting that staging practices or surgical practices which might cause stage migration did not change in the time frame of the study nor was it impacted by the cyber-attack on the Irish Health Service Executive in May 2021 [28].

### Statistical analysis

The control group was separately statistically compared to each study group for each variable above using IBM SPSS Statistics Version 29.0.1.0 (171) [29]. The continuous variables of age and tumour size were assessed for normality both visually using histograms, normal and detrended normal Q–Q plots and numerically using Shapiro–Wilk and Kolmogorov–Smirnov tests. Neither was normally distributed, and therefore, the non-parametric Mann–Whitney *U*-test was used to compare groups.

Categorical variables such as nodal status, pathological stage, hormone receptor status, histological grade, NAC and adjuvant therapies, operation type, and presence or absence of LVI were reported as figures and percentages. Groups were compared using the Pearson chi-square test of independence and Fisher’s exact test where appropriate. A *p*-value of < 0.05 deemed a result statistically significant.

This retrospective study was approved by the Clinical Research and Ethics Committee for the Cork Teaching Hospitals (CREC).

## Results

### Stage IV presentations

**Fig. 1:**
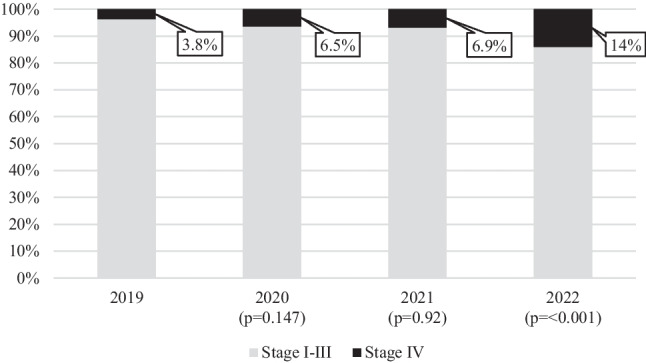
Bar chart illustrating the percentage of new breast cancer patients with stage IV disease at presentation compared to stages I–III (*y*-axis) from years 2019 to 2022 (*x*-axis)

A total of 1416 patients were diagnosed with breast cancer during the period studied with 370 cases in 2019 (control group), 341 cases in 2020, 333 cases in 2021, and 372 cases in 2022 (pandemic study groups). The percentage of breast cancers diagnosed which were metastatic at presentation was 3.8% in 2019. This significantly increased in 2022 to 14% (*p* ≤ 0.001). The increase in metastatic presentations in 2020 [6.5% vs 3.8% (*p* = 0.147)] and 2021 [6.9% vs 3.8% (*p* = 0.92)] as compared to 2019 were not statistically significant (Fig. 1).

### Neoadjuvant chemotherapy, adjuvant therapies, and axillary surgery

A total of 1130 patients who were consecutively surgically treated for invasive breast cancer from 2019 to 2022 were identified. This included 312 patients in 2019, 272 patients in 2020, 256 patients in 2021, and 290 patients in 2022. In 2019, 20.2% of these surgical patients underwent neoadjuvant chemotherapy (NAC). This percentage did not differ significantly in 2020 [18.4% vs 20.2% (*p* = 0.167)], 2021 [21.9% vs 20.2% (*p* = 0.7)], or 2022 [17.3% vs 20.2% (*p* = 0.423)].

Assessing adjuvant chemotherapy rates, 25.3% of the pre-pandemic 2019 cohort were treated with a non-significant increase in 2020 [30.2% vs 25.3% (*p* = 0.227)], no difference in 2021 [26% vs 25.3% (*p* = 0.893)], and a non-significant decrease in 2022 [20% vs 25.3% (*p* = 0.176)]. A similar trend was observed in adjuvant radiotherapy rates with 66.3% of patients treated in 2019. This significantly increased in 2020 [82.6% vs 66.3% (*p* ≤ 0.001)], did not differ in 2021 [73.4% vs 66.3% (*p* = 0.83)] and significantly decreased in 2022 [54.8% vs 66.3% (*p* = 0.005)]. When numbers of patients who underwent both adjuvant chemotherapy and radiotherapy were assessed, 20.2% of patients in 2019 had been treated. This showed non-significant increases in 2020 [26.5% vs 20.2% (*p* = 0.09)] and 2021 [23.8% vs 20.2% (*p* = 0.346)] and a significant decrease in 2022 [13.1% vs 20.2% (*p* = 0.027)].

The overall percentage of patients who had axillary surgery (either axillary node clearance (ANC) or sentinel lymph node biopsy (SLNB)) was 82.7% in 2019. This increased significantly during the pandemic to 90.4% in 2020 (*p* = 0.009) and 90.6% in 2021 (*p* = 0.009) and returned to pre-pandemic levels at 83.8% in 2022 (*p* = 0.801). Rates of ANC were 17% in 2019 and did not change significantly in 2020 [21% vs 17.3% (*p* = 0.365)], 2021 [21.9% vs 17.3% (*p* = 0.206)], or 2022 [17.3% vs 17.0% (*p* = 1)]. Similarly, rates of SLNB did not change significantly from 65.4% in 2019, during 2020 [69.9% vs 65.4% (*p* = 0.289)], 2021 [68.8% vs 65.4% (*p* = 0.448)], or 2022 [66.6% vs 65.4% (*p* = 0.829)]. These findings are summarised in Table 1.

**Table 1:** Rates of neoadjuvant chemotherapy, adjuvant therapies, axillary nodal clearance, and sentinel lymph node biopsy in patients who underwent surgery for invasive breast cancer before and after the onset of COVID-19

**Characteristics**	**Groups**			
	**2019 (control)**	**2020**	**2021**	**2022**
**Sample size**	*n* = 312	*n* = 272	*n* = 256	*n* = 289
**Neoadjuvant chemotherapy**	63 (20.2%)	42 (18.4%); (*p* = 0.167)	56 (21.9%); (*p* = 0.7)	50 (17.3%); (*p* = 0.423)
**Adjuvant chemotherapy**	79 (25.3%)	82 (30.1%); (*p* = 0.227)	67 (26.2%); (*p* = 0.843)	59 (20.3%); (*p* = 0.176)
**Adjuvant radiotherapy**	207 (66.3%)	222 (82.6%); **(*p* = < 0.001)**	188 (73.4%); (*p* = 0.83)	159 (55%); **(*p* = 0.005)**
**Adjuvant chemoradiotherapy**	63 (20.2%)	72 (26.5%); (*p* = 0.09)	61 (24.8%); (*p* = 0.346)	38 (13%); **(*p* = 0.027)**
**Axillary nodal clearance (ANC)**	54 (17.3%)	56 (20.6%); (*p* = 0.365)	56 (21.9%); (*p* = 0.206)	50 (17.0%); (*p* = 1)
**Sentinel lymph node biopsy (SLNB)**	204 (65.4%)	190 (69.9%); (*p* = 0.289)	176 (68.8%); (*p* = 0.448)	193 (67%); (*p* = 0.829)
**Axillary surgery (ANC or SLNB or both)**	366 (82.7%)	246 (90.4%); **(*p* = 0.009)**	232 (90.6%); **(*p* = 0.009)**	243 (83.8%); (*p* = 0.801)

The *p*-values in bold indicate statistically significant findings

### Nodal metastases

On assessing nodal status in patients (including the NAC cohort), 34.9% of the control patients (March to December 2019) were deemed lymph node positive on pathological staging. This percentage increased during the same period in 2020 [39.9% vs 34.9% (*p* = 0.35)] and 2021 [40.5% vs 34.9% (*p* = 0.285)], but these increases did not reach significance. There was no difference in nodal positivity rates in the 2022 group compared to 2019 [33.8% vs 34.9% (*p* = 0.889)]. These findings are summarised in Table 2.

**Table 2:** Rates of pathological nodal metastases in patients who underwent surgery for invasive breast cancer compared by year group before and after the onset of COVID-19

**Treatment groups**	**Year groups**			
	**2019 (control)**	**2020**	**2021**	**2022**
**Nodal positivity in patients who did not receive NAC**	66/175 (38.2%)	62/155 (41.1%); (*p* = 0.593)	68/158 (44.7%); (*p* = 0.229)	54/171 (34.6%); (*p* = 0.506)
**Nodal positivity in patients who received NAC**	8/43 (18.6%)	15/38 (39.5%); (*p* = 0.067)	13/42 (31.0%); (*p* = 0.285)	19/45 (42.2%); **(*p* = 0.03)**
**Nodal positivity in all surgical patients (NAC and no NAC)**	74/212 (34.9%)	77/193 (39.9%); (*p* = 0.35)	81/200 (40.5%); (*p* = 0.285)	73/216 (33.8%); (*p* = 0.889)

The *p*-values in bold indicate statistically significant findings

**Fig. 2:**
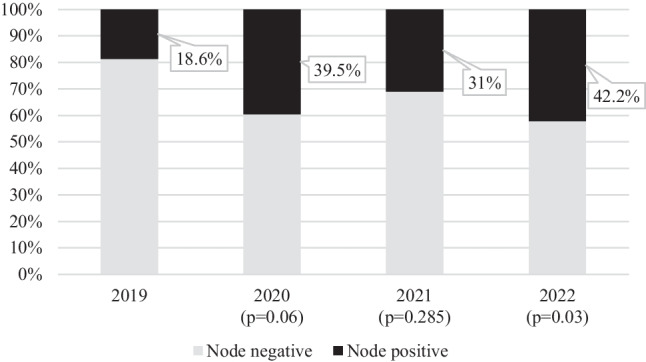
Bar chart illustrating the percentage of surgical patients who were lymph node positive on pathological staging after undergoing NAC in 2019 (*n* = 43), 2020 (*n* = 42), 2021 (*n* = 42), and 2022 (*n* = 45)

The pathological nodal status of patients who underwent surgery without NAC for invasive breast cancer was assessed separately with 38.2% of patients positive for nodal metastases in 2019. This showed non-significant increases in 2020 [41.1% vs 38.2% (*p* = 0.593)] and 2021 [44.7% vs 38.2% (*p* = 0.229)] and a non-significant decrease in 2022 [34.6% vs 38.2% (*p* = 0.506)] (Table 2). When the pathological nodal staging of only the patients who underwent NAC was assessed, 18.6% of the 2019 group were deemed node-positive. This percentage increased in the 2020 [39.5% vs 18.6% (*p* = 0.067)] and 2021 [31% vs 18.6% (*p* = 0.285)] groups without reaching significance. However, it was significantly increased in the 2022 group [18.6% vs 42.2% (*p* = 0.03)] (Table 2 and Fig. 2).

### Tumour histopathological characteristics

A total of 659 patients who were consecutively surgically treated upfront for invasive breast cancer were identified. The 175 patients in the pre-pandemic control group (March to December 2019) were separately compared to 155 patients in the first pandemic study group (March to December 2020), 158 in the second pandemic study group (March to December 2021), and 171 in the third pandemic study group (March to December 2022). The median age at diagnosis of breast cancer did not vary between the 2019 group when compared to the 2020 group [60 (31–87) vs 57 (35–85) (*p* = 0.170)], the 2021 group [60 vs 60 (22–84) (*p* = 0.791)], or the 2022 group [60 vs 60 (28–90) (*p* = 0.294)]. The clinical and pathological characteristics of the four groups are summarised in Table 3.

**Table 3:** Demographic, clinical, and pathological characteristics of the surgical groups

**Characteristics**	**Groups**			
	**2019 (control)**	**2020**	**2021**	**2022**
**Sample size**	*n* = 175	*n* = 155	*n* = 158	*n* = 171
**Age at diagnosis** (median)	60 (31–87)	57 (35–85); (*p* = 0.17)	60 (22 – 84); (*p* = 0.791)	60 (28–90); (*p* = 0.294)
**Tumour size** (median)	2.4 cm	2.6 cm; (*p* = 0.098)	2.4 cm; (*p* = 0.791)	2.1 cm; (*p* = 0.34)
**Operation type**				
Mastectomy	54 (30.9%)	53 (34.3%)	46 (29.1%)	50 (29.2%)
BCS	121 (69.1%)	102 (65.8%); (*p* = 0.518)	112 (70.9%); (*p* = 0.729)	121 (70.8%); (*p* = 0.743)
**Histological grade**				
1	5 (3.0%)	6 (3.9%)	5 (3.2%)	10 (5.9%)
2	93 (55%)	61 (39.9%)	73 (46.5%)	89 (52.7%)
3	71 (42%)	86 (56.2%); **(*p* = 0.025)**	79 (50.3%); (*p* = 0.302)	70 (41.4%); (*p* = 0.414)
**LVI**				
Positive	75 (43.1%)	78 (50.3%)	86 (54.4%)	66 (38.6%)
Negative	99 (56.9%)	77 (49.7%); (*p* = 0.19)	72 (45.6%); **(*p* = 0.039)**	105 (61.4%); (*p* = 0.458)
**ER status**				
Positive	156 (91.8%)	134 (87%)	134 (84.8%)	149 (88.2%)
Negative	14 (8.2%)	20 (13%); (*p* = 0.163)	24 (15.2%); **(*p* = 0.049)**	20 (11.8%); (*p* = 0.356);
**PR status**				
Positive	134 (78.8%)	114 (74%)	119 (75.3%)	133 (78.7%)
Negative	36 (21.2%)	40 (26%); (*p* = 0.309)	39 (24.4%); (*p* = 0.45)	36 (21.3%); (*p* = 0.27)
**HER2 status**				
Positive	13 (7.8%)	17 (11%)	11 (7%)	16 (9.5%)
Negative	154 (92.2%)	137 (89%); (*p* = 0.312)	147 (93%); (*p* = 0.777)	153 (90.5%); (*p* = 0.583)
**Pathological stage**				
1	59 (34.4%)	46 (30.3%)	48 (31.4%)	67 (42.2%)
2	86 (50%)	76 (50%)	77 (50.3%)	73 (46.2%)
3	27 (15.7%)	29 (19.1%); (*p* = 0.558)	28 (18.3%); (*p* = 0.765)	17 (10.8%); (*p* = 0.235)
**Histological subtype**				
IDC	116 (66.3%)	124 (80%)	128 (81%)	116 (68.2%)
ILC	38 (21.7%)	20 (12.9%)	16 (10.1%)	30 (17.6%)
Mixed ILC/IDC	5 (2.9%)	4 (2.6%)	5 (3.2%)	9 (5.3%)
Other	16 (9.1%)	7 (4.5%); **(*p* = 0.04)**	9 (5.7%); **(*p* = 0.014)**	15 (8.8%); (*p* = 0.563)

The *p*-values in bold indicate statistically significant findings

Median pathological tumour size was 0.2 cm larger in 2020 as compared to the 2019 control group; however, this difference was not statistically significant (2.6 cm vs 2.4 cm, *p* = 0.098). There was no difference in median tumour size between the control group and the 2021 and 2022 groups, respectively, [2.4 cm vs 2.4 cm (*p* = 0.791), 2.1 cm vs 2.4 cm (*p* = 0.34)]. The percentage of BCS performed decreased slightly in 2020 as compared to 2019 (65.8% vs 69.1%, *p* = 0.518), but this did not reach statistical significance. There was no difference in the percentage of BCS performed in 2021 or 2022 as compared to 2019 [70.9% vs 69.1% (*p* = 0.729), 70.8% vs 69.1% (*p* = 0.743)].

In terms of histological tumour characteristics, the 2020 group had a significantly higher percentage of grade 3 tumours as compared to 2019 (56.2% vs 42.0%, *p* = 0.025) and a lower percentage of grade 2 tumours (39.1% vs 55.0%). The higher percentage of grade 3 tumours in 2021 as compared to the 2019 group did not reach statistical significance (50.3% vs 42.0%, *p* = 0.302). There was no difference between the 2019 and 2022 groups in grade (*p* = 0.414). There was no significant difference between the 2019 group and the 2020 or 2022 groups in the percentage of tumours that were positive for LVI [50.3% vs 43.1%, (*p* = 0.190), 38.6% vs 43.1% (*p* = 0.458)]. However, the 2021 group did have a higher percentage of LVI as compared to 2019 (54.4% vs 43.1%, *p* = 0.039).

The percentage of ER positivity was lower in the 2021 group as compared to the 2019 group (84.8% vs 91.8%, *p* = 0.049), with no significant difference between the 2019 group and the 2020 or 2022 groups [87.0% vs 91.8% (*p* = 0.163), 88.2% vs 91.8% (*p* = 0.36)]. There was no difference between the 2019 group and any other group in the percentage of PR positivity [74.0% vs 78.8% (*p* = 0.31), 74.3% vs 78.8% (*p* = 0.45), 78.7% vs 78.8% (*p* = 0.27)] or HER-2 positivity [11.0% vs 7.8% (*p* = 0.312), 7.0% vs 7.8% (*p* = 0.78), 9.5% vs 7.8% (*p* = 0.58)]. The distribution of breast cancer subtype differed between the 2019 group and the 2020 and 2021 groups with a higher percentage of IDC and a lower percentage of ILC diagnosed in the latter [IDC, 80.0% vs 66.3% (*p* = 0.04), 81.0% vs 66.3% (*p* = 0.014); ILC, 12.9% vs 21.7%, 10.1% vs 21.7%]. The subtype distribution did not significantly differ between the 2019 and 2022 groups (*p* = 0.563).

The reduced percentage of early-stage breast cancers diagnosed in the 2020 and 2021 groups as compared to the 2019 group was not statistically significant [80.3% vs 84.3% (*p* = 0.596), 81.7% vs 84.3% (*p* = 0.532)]. There was no significant difference in the percentage of early breast cancers diagnosed between the 2019 and 2022 groups (88.6% vs 84.3%, *p* = 0.322).

## Discussion

The COVID-19 pandemic impacted the management, quality of life, and emotional well-being of symptomatic and screen-detected breast cancer patients in Ireland [24, 30, 31]. The present retrospective cohort study analysed institutional data from symptomatic breast cancer patients across the pandemic and post-pandemic period. It has demonstrated a significant increase in the proportion of new metastatic breast cancer presentations in 2022 compared to pre-pandemic levels. It has also demonstrated increases in the grade of tumour and percentage of tumours with lymphovascular invasion, changes in rates of adjuvant therapies, and changes in the hormonal profile and histological subtype of tumours diagnosed during the pandemic which returned to expected levels in 2022. Interestingly, in surgically treated patients, no significant change was demonstrated in the pathological stage, nodal positivity, tumour size, or BCS to mastectomy ratio.

Several published studies have hypothesised that patients presented with more advanced breast cancers during the pandemic. Heterogeneity in results exists amongst these studies, with many reporting more advanced breast cancer diagnoses in terms of higher numbers of advanced-stage cancers [19, 20], fewer numbers of early-stage cancers [21, 32, 33], increased tumour size [34], and higher levels of node-positive disease [18, 35], and others finding no significant difference [36–38]. In addition, many studies reported a decrease in the ratio of BCS to mastectomy, reflecting the lower numbers of early-stage breast cancers being diagnosed [39, 40]. The lower number of early-stage tumours and larger tumour size in surgical patients during the pandemic, and the decrease in the numbers of BCS performed, did not reach statistical significance in this study. This may reflect the fact that rapid access to symptomatic breast clinics remained operational during the pandemic, with the initial drop in attendance in March 2020 recovering within 2 months [41]. It could also reflect a relatively small-sized study performed in a single institution.

However, the increase in new metastatic breast cancer presentations in 2022 as compared to pre-pandemic levels is significant. The lag in this noted increase from the start of the pandemic in early 2020 until 2022 may reflect the effect of interruptions to the national breast cancer screening programme during the pandemic, leading to cancers which may have been diagnosed earlier on screening prior to the pandemic presenting as advanced symptomatic disease. Patient delays in presenting for evaluation of symptoms and other factors including the more aggressive histopathological profile of tumours diagnosed during the pandemic study may also have contributed to an increase in new metastatic presentations. Few existing studies have included data from 2022, but a similar increase in metastatic breast cancer presentations in 2020/2021 pandemic cohorts has been demonstrated in numerous other countries [19, 42].

A vital prognostic factor in patients with invasive breast cancer is axillary nodal status [43]. Other studies assessing rates of nodal positivity in pre-pandemic and pandemic cohorts have produced varying results, with several finding increased rates of nodal metastases after the onset of COVID-19 [18, 19, 44], and others reporting no change [45, 46]. The present study found no significant increase overall in rates of nodal metastases in invasive breast cancer patients across the pandemic period, although it is interesting to note a significant increase in pathological nodal positivity within the cohort who underwent NAC in 2022 as compared to pre-pandemic cohorts. This may reflect the higher rates of stage IV disease noted in 2022 as discussed above; however, the small sample size of patients who underwent NAC limits any conclusions that can be drawn. Rates of ANC and SLNB remained stable across the pandemic period, although the overall number of patients who had either or both increased in 2020 and 2021. Contributory factors to this may have included the increased histological grade and increased number of ER tumours diagnosed during these years [47] or an increased number of patients requiring both SLNB and subsequent ANC.

Patterns in the use of NAC, adjuvant chemotherapy and adjuvant radiotherapy varied significantly by country during the pandemic based on differing local guidelines [25]. The present study has not demonstrated any change in rates of NAC or adjuvant chemotherapy across the pandemic period. There was a significant increase in patients receiving adjuvant radiotherapy in 2020 despite no increase in BCS; this could reflect non-significant increases in median tumour size and nodal metastases in this pandemic cohort. There was a significant decrease in adjuvant radiotherapy rates in 2022, where the increase in stage IV diagnoses may have contributed to fewer patients being eligible for this treatment. Relatively few studies have assessed changes to adjuvant radiotherapy rates across the pandemic, with an Italian study reporting a similar increase in 2020 and others reporting changes in treatment regimens [25, 48].

Histological grade and the presence of LVI and hormone receptor status are well-recognised as prognostic indicators in breast cancer [49–51], with grade and receptor status used as factors in organising breast carcinomas into prognostic stage groups in the widely used 8th edition of the AJCC manual [26]. This study demonstrated a significant increase in the percentage of grade three tumours diagnosed in 2020 and a non-significant increase in 2021, with numbers in 2022 returning to 2019 levels. A similar retrospective cohort study in Italy looking at both symptomatic and screen-detected cases surgically treated in one centre also found an increase in grades two and three tumours during the pandemic [52]; however, no similar study investigating only symptomatic cases was identified in the literature review.

The percentage of tumours negative for ER was also significantly increased in the 2021 group; ER-negative tumours are associated with higher histological grade and a worse prognosis than ER-positive cases [53], suggesting that symptomatic cancers of a more aggressive phenotype were diagnosed in our institution during the pandemic. In addition, proportionally fewer cases of ILC and more IDC were diagnosed in both 2020 and 2021, which may reflect the increased likelihood of experiencing symptoms with IDC compared to ILC during a period when breast cancer screening was paused [54]. The percentage of tumours featuring LVI on histopathological assessment in this study was also significantly higher in 2021 as compared to the 2019 control group. Several other published studies evaluating only screen-detected or both screen-detected and symptomatic breast cancers found no significant difference in histological subtype or hormonal status [22, 55].

As breast cancer diagnostic and treatment pathways return to normal, the impact on longer-term outcomes such as recurrence and survival for patients newly diagnosed with breast cancer during the pandemic is still unclear. Delays in patients with breast symptoms presenting to primary care during the pandemic period due to anxieties around COVID-19 and the pausing of the National Breast Screening Programme during lockdown periods may have led to the increase in metastatic presentations evident in the 2022 data and symptomatic cancers with less favourable prognostic factors being diagnosed since the pandemic. This highlights the importance of public health messaging around cancer as well as continued access to screening, diagnostic, and treatment pathways for breast cancer during public health crises. Long-term follow-up and population-level modelling studies are necessary to fully understand the prognostic impact of the pandemic on breast cancer patients and to assess the trend of increased metastatic presentations and mortality in breast cancer patients in the coming years.

This study has some important limitations. The retrospective nature of the study is inherently limiting, as is the inclusion of a single institution and therefore a relatively small sample. In addition, the inclusion of only symptomatic breast cancer patients limits the generalizability of the findings. However, it is a relevant study in being one of the few to compare symptomatic breast cancer presentations across the COVID-19 period over a range of histopathological variables.

## Conclusion

Symptomatic breast cancers diagnosed during the COVID-19 pandemic demonstrated an increase in metastatic disease at presentation along with higher histological grade, more frequent lymphovascular invasion, and differing hormone receptor and histological subtypes when compared to pre-pandemic cases. Presentations did not differ significantly in nodal status, tumour size, or type of operation performed. Rates of adjuvant radiotherapy and axillary surgery increased early in the pandemic, which may reflect more advanced cancer presentations. Longer-term follow-up studies with close evaluation of rates of metastatic breast cancer presentations and the related increased mortality in coming years are essential in planning for future public health crises and public health messaging. Extra support and resources for breast cancer treatment pathways to deal with the burden of the current more advanced disease presentations may be necessary.

## Data Availability

All data supporting the findings of this study are contained in this report.
